# Comparative analysis of neurofilaments and biomarkers of muscular damage in amyotrophic lateral sclerosis

**DOI:** 10.1093/braincomms/fcae288

**Published:** 2024-08-26

**Authors:** Maximilian Vidovic, Hanna Sophie Lapp, Constanze Weber, Lydia Plitzko, Michael Seifert, Petra Steinacker, Markus Otto, Andreas Hermann, René Günther

**Affiliations:** Department of Neurology, University Hospital Carl Gustav Carus, Technische Universität Dresden, Dresden 01307 Germany; Department of Neurology, University Hospital Carl Gustav Carus, Technische Universität Dresden, Dresden 01307 Germany; Department of Neurology, University Hospital Carl Gustav Carus, Technische Universität Dresden, Dresden 01307 Germany; Department of Neurology, University Hospital Carl Gustav Carus, Technische Universität Dresden, Dresden 01307 Germany; Carl Gustav Carus Faculty of Medicine, Institute for Medical Informatics and Biometry (IMB), Technische Universität Dresden, Dresden 01307, Germany; Department of Neurology, Martin-Luther-University of Halle-Wittenberg, Halle (Saale) 06120, Germany; Department of Neurology, Martin-Luther-University of Halle-Wittenberg, Halle (Saale) 06120, Germany; Translational Neurodegeneration Section ‘Albrecht Kossel’, Department of Neurology, University Medical Center Rostock, University of Rostock, Rostock 18147, Germany; German Center for Neurodegenerative Diseases (DZNE), Site Rostock/Greifswald, Rostock 18147, Germany; Department of Neurology, University Hospital Carl Gustav Carus, Technische Universität Dresden, Dresden 01307 Germany; German Center for Neurodegenerative Diseases (DZNE), Site Dresden, Dresden 01307, Germany

**Keywords:** amyotrophic lateral sclerosis, ALS, biomarkers, neurofilaments, biomarkers of muscular damage

## Abstract

Diagnosis of the fatal neurodegenerative disease amyotrophic lateral sclerosis is challenging. Neurofilaments, indicative of neuronal damage, along with creatine kinase, creatinine, myoglobin, and troponin T, representing muscular damage, have been identified as promising fluid biomarkers. This study aims to comprehensively assess and compare their diagnostic and prognostic potential in a ‘real-world’ cohort of patients with amyotrophic lateral sclerosis. About 77 patients with amyotrophic lateral sclerosis and its clinical variants, and 26 age- and sex-matched controls with various neuromuscular and neurodegenerative diseases, were retrospectively included in this monocentric, cross-sectional study. Neurofilaments in cerebrospinal fluid and biomarkers of muscular damage in serum were measured and correlated with demographic features, motor function, survival time, clinical phenotypes, and the extent of upper and lower motor neuron involvement. Neurofilament, myoglobin, and troponin T concentrations were higher in patients with amyotrophic lateral sclerosis compared to disease controls. Higher neurofilament levels correlated with lower motor function and faster disease progression rate, while higher creatine kinase and creatinine concentrations were linked to preserved motor function. In contrast, troponin T elevation indicated poorer fine and gross motor functions. Increased neurofilament levels were associated with shorter survival, whereas biomarkers of muscular damage lacked survival correlation. Neurofilament concentrations were higher in classical amyotrophic lateral sclerosis than in progressive muscular atrophy, while myoglobin and troponin T levels were elevated in progressive muscular atrophy compared to primary lateral sclerosis. Neurofilaments were predominantly linked to upper motor neuron involvement. Our findings confirmed the robust diagnostic and prognostic value of neurofilaments in amyotrophic lateral sclerosis. Elevated neurofilament concentrations were associated with higher disease severity, faster disease progression, shorter survival, and predominant upper motor neuron degeneration. Biomarkers of muscular damage were inferior in distinguishing amyotrophic lateral sclerosis from other neuromuscular and neurodegenerative diseases. However, they may serve as complementary biomarkers and support in discriminating clinical variants of amyotrophic lateral sclerosis.

## Teaser

This cross-sectional, retrospective study compares neurofilaments and muscular biomarkers in amyotrophic lateral sclerosis. Neurofilaments show robust diagnostic and prognostic potential, correlating with disease severity, progression, survival, and upper motor neuron involvement. Biomarkers of muscular damage are inferior but may be complementary and aid in clinical variant discrimination.

## Graphical Abstract

**Graphical Abstract:**
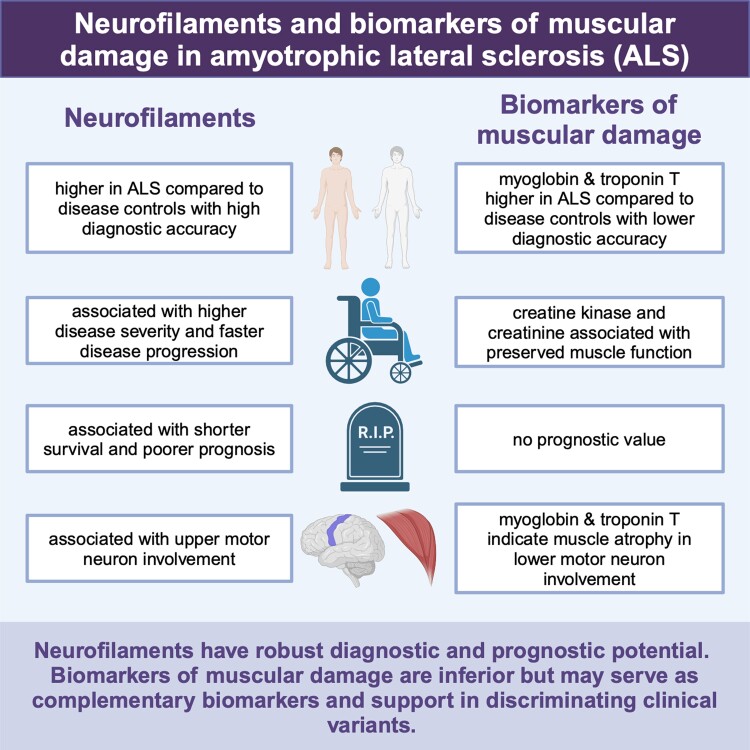

## Introduction

Amyotrophic lateral sclerosis (ALS) is a fatal neurodegenerative disease characterized by progressive loss of motor neurons in the brain and spinal cord, leading to weakness of the voluntary muscle system and ultimately to death due to respiratory failure.^1^

For years, diagnosis of ALS has primarily encompassed clinical and electrophysiological findings. Given a survival expectancy of 2–4 years,^2^ diagnostic delay is a major concern. Easily accessible biomarkers with high diagnostic accuracy would facilitate not only the early detection of ALS but also the monitoring of disease progression, treatment management, enrollment of patients for new clinical trials, even in pre-symptomatic stages, and evaluation of the efficacy of novel disease-modifying agents.^2-5^ Given the continuous emergence of new therapeutic approaches for ALS, there is an urgent need for improved accessibility and a deeper understanding of the interpretation of biomarkers within the routine clinical context. Additionally, it is crucial to carefully assess the strengths and limitations of these biomarkers.

Neurofilaments (Nf), the predominant structural components of neuronal cytoskeleton, have garnered significant attention as potential indicators of neuronal damage and degeneration in ALS. Elevated levels, particularly phosphorylated neurofilament heavy chain (pNfH) and neurofilament light chain (NfL), have been consistently observed in cerebrospinal fluid (CSF) and blood samples of patients with ALS, reflecting the axonal damage occurring in the disease.^6-9^ These Nf were reported as being strong predictors not only for motor impairment, but also for disease progression and survival.^7,10,11^ Recent studies also revealed the therapeutic responsiveness of NfL to the intrathecally administered antisense oligonucleotide (ASO) tofersen in patients with ALS and mutations in the *superoxide dismutase 1* (*SOD1*) gene.^12-15^ Tofersen has been granted accelerated approval by the U.S. Food and Drug Administration (FDA) in April 2023 and is available via expanded access programmes outside the USA.^16^

Concomitantly, fluid biomarkers of muscular damage (BMD) have also emerged as complementary indicators of ALS pathology. The hallmark feature of ALS, motor neuron degeneration, inevitably leads to denervation of skeletal muscles, resulting in profound muscle wasting. This process is associated with the release of various intramuscular proteins into the bloodstream. Elevated serum levels of these markers have been detected in patients with ALS, providing valuable insights into the extent of muscle damage and potentially serving as additional diagnostic and prognostic indicators. Creatine kinase (CK) and creatinine (Crn) are part of muscle energy metabolism and are affected in ALS,^17-20^ as they reflect muscle mass and muscle integrity. In adult patients with motor neuron disease 5q-associated spinal muscular atrophy (SMA), primarily affecting lower motor neurons (LMN), both biomarkers have shown potential as predictors of a treatment course with the ASO nusinersen in a retrospective study.^21^ In ALS, CK was reported to be a positive prognostic factor and associated with spinal onset^22,23^ and it was also postulated to be elevated in the early stages of ALS preceding muscle atrophy.^17^ Furthermore, higher CK levels were associated with a higher burden of LMN dysfunction than of upper motor neuron (UMN) dysfunction, and a lower disease progression rate.^18^ Also for Crn, associations with functional motor outcome and survival in ALS were reported.^24^ Myoglobin (Mb) is a cytoplasmic hemoprotein within cardiac and striated muscles storing oxygen and releasing it upon mitochondrial demands.^25,26^ Higher Mb levels were linked to a slower disease progression in ALS assuming it to be a protective factor and indicating higher muscular metabolic reservoir.^20^

The highly sensitive cardiac marker troponin T (TnT) has also recently been described within the context of ALS, SMA, and other neuromuscular diseases, serving as a promising fluid biomarker for muscle wasting.^27-29^ In a large cohort study, TnT was elevated in more than 60% of patients with ALS. Moreover, it is assumed to serve as a proxy of LMN or skeletal muscle involvement, correlating with disease severity and increasing over the disease course.^30^

To our knowledge, data comparing the potential and importance of biomarkers associated with neurodegeneration and muscle wasting are lacking. In the quest for reliable biomarkers in ALS, this study aims to comparably investigate the role of Nf and BMD and comprehensively evaluate their diagnostic accuracy, prognostic value, and potential implications in a ‘real-world’ setting.

## Patients and methods

### Study cohort, diagnostic assessments

A total of 77 patients with ALS and ALS variants, and 26 age- and sex-matched controls were retrospectively enrolled in this monocentric study. The controls included neuromuscular diseases mimicking ALS (adrenoleucodystrophy, *n* = 1; benign fasciculation crampus syndrome, *n* = 2; cervical or lumbar myelopathy, *n* = 3; chronic inflammatory demyelinating polyneuropathy, *n* = 4; hereditary spastic paraplegia, *n* = 5; myopathy, *n* = 2; and idiopathic focal muscle weakness, *n* = 2) and other neurodegenerative diseases (Creutzfeldt–Jakob’s disease, *n* = 1; Friedreich’s ataxia, *n* = 1; multiple system atrophy, *n* = 1; Parkinson’s disease, *n* = 2; progressive supranuclear palsy, *n* = 1; and cerebellar atrophy, *n* = 1). Patients with ALS or ALS variants were included based on the diagnostic criteria referring to Gold Coast criteria^31^ and the availability of clinical and laboratory assessments. Clinical phenotypes of ALS were defined as follows: classical form of ALS (cALS) with spinal or bulbar onset and combined UMN and LMN impairment, progressive muscle atrophy (PMA) with clinically isolated LMN impairment, and primary lateral sclerosis (PLS) with clinically isolated UMN impairment.^2,4^ Number of affected regions with UMN (bulbar, cervical, and lumbar region) or LMN (bulbar, cervical, thoracic, and lumbar region) involvement were clinically evaluated according to the revised El Escorial criteria.^32^ The control group included randomly selected patients with ALS mimics and other neurodegenerative diseases. All patients were examined as part of the routine outpatient and inpatient care at the Department of Neurology, University Hospital Carl Gustav Carus, Dresden, Germany, from February 2018 to December 2023. Patients with acute kidney injury, chronic kidney disease, myocardial ischaemia, and coronary artery disease were excluded. The study was approved by the local ethics committee (BO-EK-545122022).

The demographic and clinical data of patients were collected including age, sex, body mass index (BMI), disease duration, site of onset, ALS-related gene mutations, forced vital capacity (FVC), and status of percutaneous endoscopic gastrostomy (PEG) tube and ventilation (invasive or non-invasive).

CSF was obtained by lumbar puncture (LP), which was performed as part of the regular diagnostic routine. NfL values were quantified at the central laboratory of the University Hospital Dresden using the UmanDiagnostics NF-light™ enzyme-linked immunosorbent assay (ELISA) test kit and pNfH values were quantified at the laboratory of the University Hospital Ulm, Germany, using BioVendor standard ELISA kits. Blood samples used for analysis were either obtained at the time point of LP or over a maximum period of 30 days before or after LP. BMD were obtained by blood samples, and all were measured at the central laboratory of the University Hospital Carl Gustav Carus, Dresden, Germany, using Roche Diagnostics cobas® *in vitro* assays.

The ALS Functional Rating Scale in its revised form (ALSFRS-R) was used to assess the functional motor status and disease severity. The ALSFRS-R consists of 12 items categorized into four subdomains: bulbar, fine motor, gross motor, and respiratory functions. The scale has a maximum of 48 points with lower scores indicating a more severe functional impairment.^33^ Disease duration was defined as the time difference between the onset of first symptoms (muscle weakness or speech/swallowing/breathing disturbances) and laboratory sampling. The ALS disease progression rate (ALS-PR) was calculated by the formula: 48—ALSFRS-R score closest to the date of laboratory sampling divided by disease duration (in months).^34^

### Statistical analysis

Statistical analysis and data visualization were performed using GraphPad Prism 10 [GraphPad Software Inc., San Diego (CA), USA] and SPSS Statistics 27 [IBM, Chicago (IL), USA].

Unless otherwise stated, Nf and BMD data and the assessed scores are presented as median ± interquartile range (IQR). Both Nf and BMD data were not normally distributed as tested by Shapiro–Wilk test (*P* < 0.001). Therefore, rank-based, non-parametric tests were used for analysing group differences.

To estimate the comparability of the study group and control group, we used Pearson’s Chi-squared test for equal distribution regarding sex and Mann–Whitney *U*-test concerning conformity of age. Mann–Whitney *U*-test was used for the comparison of biomarker values between the study group and the control group. Effect sizes were reported considering *r* = 0.10 as a weak, *r* = 0.30 as a moderate, and *r* = 0.50 as a strong effect.^35^ Receive operating characteristic (ROC) analyses with given area under the curve (AUC) values were performed for discrimination between ALS and disease controls. The Youden Index was calculated for determining the optimal cut-off value. Combined ROC analyses were calculated for significant univariate ROC analyses. Predicted probabilities given by a binomial logistic regression with Nf and BMD as covariates and disease state as the dependent variable were used. AUC values between 0.7–0.9 were referred to as moderate, ≥ 0.9 as high diagnostic accuracy.^36^ Kruskal–Wallis Test was used for the comparison of study subgroups regarding demographic features. To investigate the association between biomarker values and functional motor status scores, we correlated Nf and BMD data with clinical features using Spearman’s rank correlation coefficient (*ρ*). We corrected for age, sex, and disease duration using partial rank correlation, as these variables were suspected to be confounding factors for Nf,^7,37^ and for BMD including BMI as a covariate. A correlation coefficient of *ρ* < 0.3 was considered as a weak, *ρ* = 0.3–0.59 as a moderate, and *ρ* > 0.6 as a strong correlation (modified from^38^). Adjusted *P* values after Bonferroni correction are given. We used a one-way analysis of covariance (ANCOVA) with Bonferroni-corrected *post hoc* comparison of Nf and BMD between the clinical phenotypes, considering ALS-PR as a confounding factor, because this variable differed significantly between the examined subgroups. To meet the assumptions of ANCOVA, we subjected the data of Nf and BMD (dependent variables) to a natural logarithmic transformation.

For survival analysis, we classified the patients into two groups (low with concentration ≤ median, high with concentration > median). The event was defined as death or tracheostomy. Kaplan–Meier method with log-rank test was performed for differences between both groups. In the case of significant univariate Kaplan–Meier analysis, regression analyses including age, site of onset, and ALS-PR as prognostic covariates were calculated. Statistical significance threshold was set as *P* < 0.05 two-sided.

### Heatmap of biomarker measurements

The biomarker measurements of 77 patients with ALS and 26 disease controls were visualized by a heatmap to analyse the potential of individual biomarkers to discriminate between different disease variants and between disease and disease controls. The heatmap was created in R (R function heatmap.3) using the Euclidean distance as a distance measure between individual samples in combination with Ward's minimum variance linkage method (ward.D2)^39^ as a hierarchical cluster algorithm.

## Results

Seventy-seven adult patients with ALS and clinical ALS variants were included in the analysis. Median age was 63 years (IQR, 56.0 –75.5), and the median BMI was 24.7 kg/m^2^ (IQR, 22.0–27.0); 46.8% of the study group were female and 53.2% were male. The age- and sex-matched disease control group consisted of 26 adult patients with neuromuscular diseases mimicking ALS and other neurodegenerative diseases. In the disease control group, median age was 64 (IQR 59.0–72.0). 30.8% of the disease control group were female and 69.2% were male. No significant differences were found between the groups regarding age (*U* = 961.0, *Z* = −0.304, and *P* = 0.761) and sex (*χ²*(1) = 2.029, *P* = 0.176). Details of study group characteristics are summarized in Table 1.

**Table 1:** Demographic features of patients with ALS and disease controls

	ALS (*n* = 77)	Disease controls (*n* = 26)
Age (years)		
Median (IQR)	63 (56.0–75.5)	64 (59.0–72.0)
Sex, *n* (%)		
Male	41 (53.2)	18 (69.2)
Female	36 (46.8)	8 (30.8)
BMI (kg/m^2^)		-
Median (IQR)	24.7 (22.0–27.0)	
Disease duration (months)		-
Median (IQR)	12 (8.0–22.5)	
Gene mutation, *n* (%)		-
None	70 (90.9)	
*SOD1*	5 (6.5)	
*FUS*	1 (1.3)	
*SQSTM1*	1 (1.3)	
Site of onset, *n* (%)		-
Spinal	52 (67.5)	
Bulbar	25 (32.5)	
Age at disease onset (years)		-
Median (IQR)	63 (55.0–72.5)	
ALS clinical phenotype, *n* (%)		-
cALS	63 (81.8)	
PMA	11 (15.6)	
PLS	3 (3.9)	
ALSFRS-R total (0–48 points)		-
Median (IQR)	40 (36.0–43.5)	
ALS-PR		-
Median (IQR)	0.5 (0.26–1.24)	
PEG, *n* (%)		-
Yes	5 (6.5)	
No	72 (93.5)	
Ventilation, *n* (%)		-
Yes	9 (11.7)	
Invasive	5 (6.5)	
Non-invasive	4 (5.2)	
No	68 (88.3)	
Absolute FVC (ml)		-
Median (IQR)	2950 (2025–3760)	
Predicted FVC (%)		-
Median (IQR)	85 (67.25–94.5)	

ALS, amyotrophic lateral sclerosis; ALSFRS-R, ALS Functional Rating Scale—revised form; ALS-PR, amyotrophic lateral sclerosis progression rate; BMI, body mass index; cALS, classical amyotrophic lateral sclerosis; *FUS*, *fused in sarcoma* gene; FVC, forced vital capacity; PEG, percutaneous endoscopic gastrostomy; PLS, primary lateral sclerosis; PMA, progressive muscular atrophy; *SOD1*, *superoxide dismutase 1* gene; *SQSTM1*, *sequestosome 1* gene.

### Discrimination between ALS and disease controls

**Figure 1:**
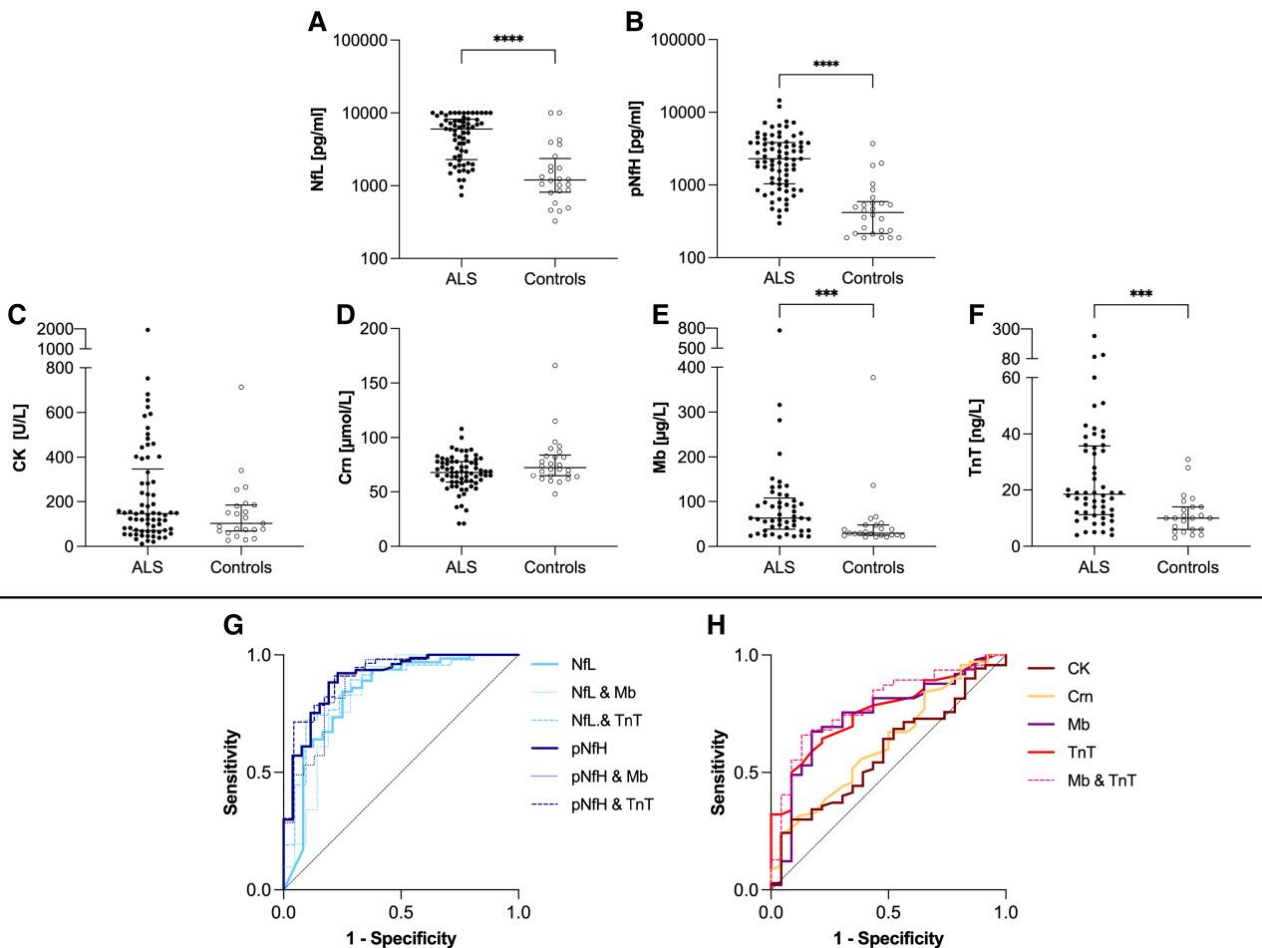
**Nf and BMD in ALS and disease controls.** Nf in ALS and disease controls **(A–B)**. BMD in ALS and disease controls (**C–F)**. Horizontal line shows median, whiskers illustrate interquartile range (0.25–0.75), and each icon represents an individual patient. Calculated by Mann–Whitney *U*-test. Significance levels: ***P < 0.001; ****P < 0.0001. ROC curves of Nf, and Nf combined with Mb or TnT for discrimination between ALS and disease controls **(G)**. ROC curves of BMD for discrimination between ALS and disease controls **(H)**. Dotted line: classifier reference line. ALS, amyotrophic lateral sclerosis; BMD, biomarkers of muscular damage; CK, creatine kinase; Crn, creatinine; Mb, myoglobin; Nf, neurofilaments; NfL, neurofilament light chain; pNfH, phosphorylated neurofilament heavy chain; TnT, troponin T.

Nf concentrations were higher in ALS than in disease controls, showing large effect sizes (NfL: *P* < 0.0001, *r* = 0.515; pNfH: *P* < 0.0001; *r* = 0.6; Fig. 1A and B, Table 2). Mb and TnT concentrations were also elevated in patients with ALS compared to disease controls. However, these differences showed only medium effect sizes (Mb: *P* = 0.0008, *r* = 0.387; TnT: *P* = 0.0002, *r* = 0.406; Fig. 1E and F, Table 3). CK and Crn concentrations of patients with ALS did not differ from the disease control group (CK: *P* = 0.235; Crn: *P* = 0.056; Fig. 1C and D, Table 3). ROC curve analysis revealed a cut-off value for NfL ≥ 1875pg/ml with a sensitivity of 84.4% and a specificity of 75.0%, and a cut-off value for pNfH ≥ 569 pg/ml with a sensitivity of 92.2% and a specificity of 76.9%. The AUC was 0.836 (*P* < 0.0001) for NfL and 0.9 (*P* < 0.0001) for pNfH (Fig. 1G). Mb yielded an optimal cut-off value of ≥ 53 µg/L with a sensitivity of 67.4% and a specificity of 82.6%. The cut-off value for TnT was 15 ng/L with a sensitivity of 64.3% and a specificity of 78.3%. The AUC was 0.741 (*P* = 0.001) for Mb and 0.76 (*P* = 0.0003) for TnT. ROC analyses of CK and Crn were not statistically significant (CK: *P* = 0.235; Crn: *P* = 0.056). ROC curves of BMD are depicted in Fig. 1H.

**Table 2:** Nf in patients with ALS and disease controls

	ALS	Disease controls	ALS versus Disease controls		
			*P* value	Effect size *r*	*Post hoc* power
**NfL [pg/ml]^a^,**	*n* = 64	*n* = 24	<0.0001***	0.515	0.99
Median	6003	1199			
(IQR)	(2288–8217)	(816–2364)			
Range	738–10 000	327–10 000			
Above ULOQ, *n* (%)	11 (17.2)	2 (8.3)			
**pNfH [pg/ml]^a^,**	*n* = 77	*n* = 26	<0.0001***	0.6	0.99
Median	2304	417.5			
(IQR)	(1041­–3827)	(215–591)			
Range	297–14 542	188–3708			
Below LLOQ, *n* (%)	–	5 (19.2)			

^a^Mann–Whitney *U*-test. *** *P* < 0.0001. Cohen’s effect sizes of *r* = 0.10, *r* = 0.30, and *r* = 0.50 as thresholds for small, medium, and large effects, respectively. ALS, amyotrophic lateral sclerosis; LLOQ, lower limit of quantitation; Nf, neurofilaments; NfL, neurofilament light chain; pNfH, phosphorylated neurofilament heavy chain; ULOQ, upper limit of quantitation.

**Table 3:** BMD in patients with ALS and disease controls

	ALS	Disease controls	ALS versus Disease controls		
			*P* value	Effect size *r*	*Post hoc* power
**CK (IU/L)^a^,**	*n* = 70	*n* = 23	0.235	0.123	0.17
Median	146.4	103.2			
(IQR)	(80.0­–347.9)	(68.4–184.8)			
range	12–1952	27–713.4			
**Crn (µmol/L)^a^,**	*n* = 70	*n* = 26	0.056	0.195	0.39
Median	68.0	72.5			
(IQR)	(59–78)	(64.8–84)			
Range	21–108	48–166			
**Mb (µg/L)^a^,**	*n* = 49	*n* = 23	0.0008**	0.387	0.89
Median	63.2	29.3			
(IQR)	(38.6–108.0)	(24.8–47.6)			
Range	21–766	21–377			
**TnT [ng/L]^a^,**	*n* = 56	*n* = 23	0.0002**	0.406	0.93
Median	18.5	10.0			
(IQR)	(11.3–35.8)	(6.0–14.0)			
Range	4–248	3–31			

^a^Mann–Whitney *U*-test. ** *P* < 0.001. Cohen’s effect sizes of *r* = 0.10, *r* = 0.30, and *r* = 0.50 as thresholds for small, medium, and large effects, respectively. ALS, amyotrophic lateral sclerosis; BMD, biomarkers of muscular damage; CK, creatine kinase; Crn, creatinine; Mb, myoglobin; TnT, troponin T.

Combining NfL and TnT to discriminate between ALS and disease controls yielded optimal diagnostic accuracy with a sensitivity of 72.3% and a specificity of 90.5%. The AUC was 0.862 (*P* < 0.0001), slightly lower than that of NfL alone. Utilizing pNfH and TnT, sensitivity was 91.1%, and specificity was 78.3%, presenting a higher specificity than that of pNfH alone. The AUC was 0.912 (*P* < 0.0001). A combination of NfL and Mb revealed an optimal sensitivity of 95.1% and a specificity of 66.7%, given an AUC of 0.829 (*P* < 0.0001). Combination pNfH and Mb revealed a similar diagnostic accuracy with a sensitivity of 91.8% and a specificity of 73.9% with an AUC of 0.879 (*P* < 0.0001). Combining both Nf with Mb, AUC was lower than that of Nf alone indicating no advantage in the diagnostic accuracy (Fig. 1G). Investigating combined testing of Mb and TnT, sensitivity was 66.0% and specificity was 87.0%. The AUC of 0.786 (*P* = 0.0001) was superior to that of Mb and TnT alone (Fig. 1H).

### Associations between biomarkers and disease severity

**Figure 2:**
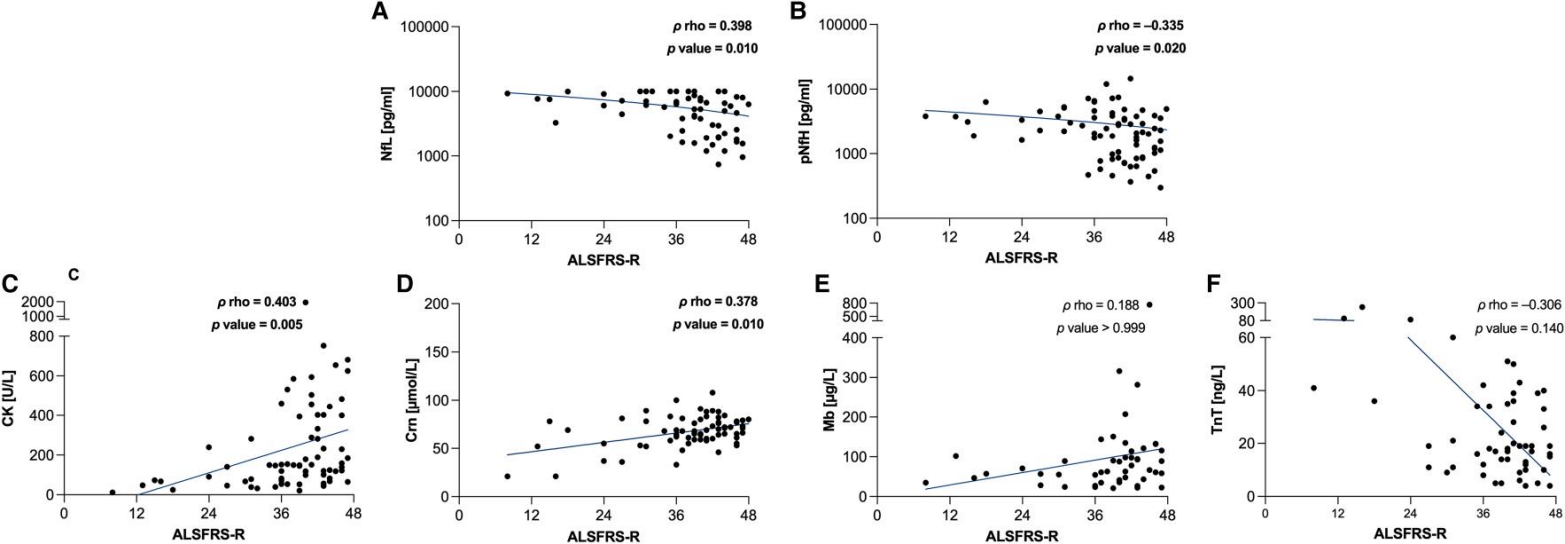
**Nf and BMD in relation to functional motor status.** Correlations between Nf and the ALSFRS-R total score **(A–B)**. Correlations between BMD and the ALSFRS-R total score **(C–F)**. Regression line in blue. Each icon represents an individual patient. Calculated by Spearman’s partial rank correlation adjusted for sex, age, BMI, and disease duration; *P* values < 0.05 considered statistically significant and marked in bold. ALS, amyotrophic lateral sclerosis; ALSFRS-R, ALS Functional Rating Scale—revised form; BMD, biomarkers of muscular damage; CK, creatine kinase; Crn, creatinine; Mb, myoglobin; Nf, neurofilaments; NfL, neurofilament light chain; pNfH, phosphorylated neurofilament heavy chain; TnT, troponin T.

After adjusting for age, sex, and disease duration, the ALSFRS-R total scores showed a moderate inverse correlation with NfL (*ρ* = −0.398, adjusted *P* = 0.01) and pNfH concentrations (*ρ* = −0.335, adjusted *P* = 0.020) (Fig. 2A and B). Regarding the ALSFRS-R subdomains, we observed moderate inverse correlations between NfL concentrations and gross motor functions (*ρ* = −0.378, adjusted *P* = 0.015), and pNfH concentrations and bulbar functions (*ρ* = −0.384, adjusted *P* = 0.005). No significant associations were found between Nf levels and the other ALSFRS-R subscores (Supplementary Table 1).

CK concentrations were moderately correlated with the ALSFRS-R total score after correction for age, sex, BMI, and disease duration (*ρ* = 0.403, adjusted *P* = 0.005). Of the ALSFRS-R subscores, CK concentrations were moderately correlated with respiratory functions (*ρ* = 0.420; adjusted *P* = 0.005). Also, Crn concentrations showed moderate correlation with the ALSFRS-R total score (*ρ* = 0.378, adjusted *P* = 0.01), and the subscores of fine motor functions (*ρ* = 0.329, adjusted *P* = 0.035) and gross motor functions (*ρ* = 0.379, adjusted *P* = 0.005). TnT concentrations were inversely correlated with ALSFRS-R fine motor (*ρ* = −0.449, adjusted *P* = 0.005) and gross motor functions (*ρ* = −0.380, adjusted *P* = 0.025) (Supplementary Table 1). However, they did not show significant correlations with the ALSFRS-R total score. Regarding Mb, no significant correlations were found. Figure 2C to F illustrates correlation plots for BMD and the ALSFRS-R total score.

### Associations between biomarkers and disease progression

Nf concentrations were moderately correlated with ALS-PR [NfL: *ρ* = 0.552, *P* < 0.001; pNfH: *ρ* = 0.511, *P* < 0.001 (Supplementary Fig. 1A and B)]. In contrast, BMD did not show any significant correlations with ALS-PR (Supplementary Fig. 1C to F).

### Associations between biomarkers and other clinical assessments

Nf concentrations were inversely correlated with disease duration (NfL: *ρ* = −0.471, *P* < 0.001; pNfH: *ρ* = −0.464, *P* < 0.001). Patients without assisted ventilation had higher CK (*P* = 0.009) and Crn levels (*P* = 0.022). Contrary, TnT levels were increased in patients with spinal onset (*P* = 0.008), with PEG (*P* = 0.038), and with assisted ventilation (*P* = 0.029). No significant correlations were detected between Nf or BMD and predicted FVC. Details are summarized in Supplementary Table 2.

### Biomarkers in relation to clinical ALS phenotypes

Sixty-three patients were phenotypically classified as cALS, whereas 11 patients were classified as PMA and 3 patients as PLS. Descriptive data of biomarker concentrations in the clinical phenotypes are summarized in Supplementary Table 3.

**Figure 3:**
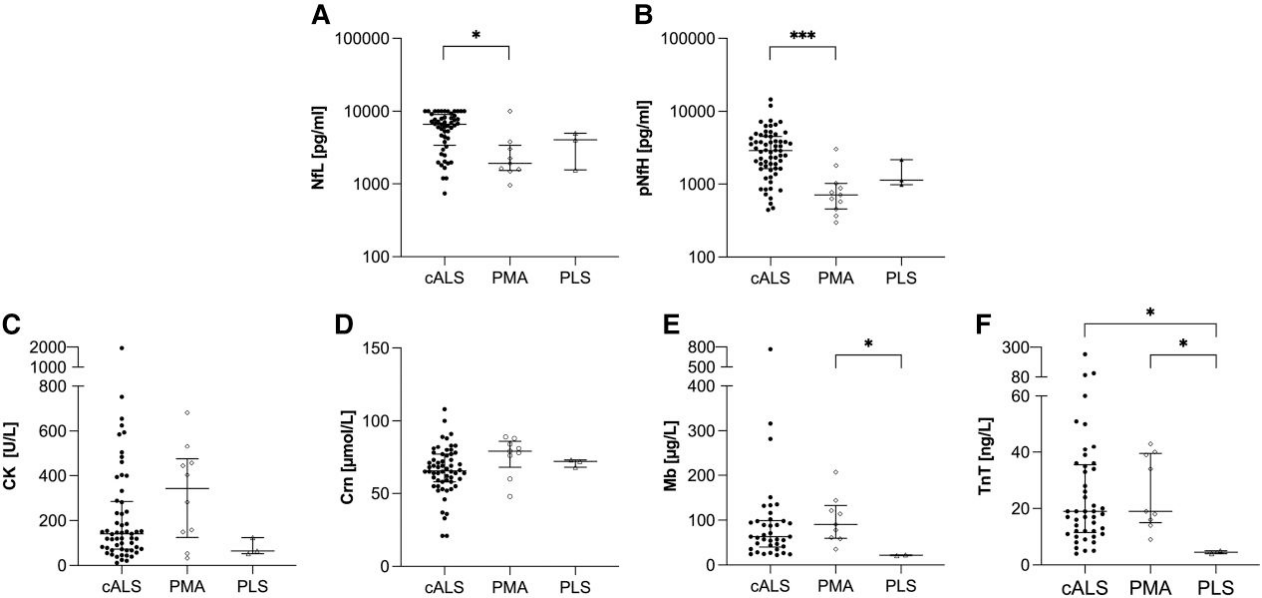
**Nf and BMD in relation to clinical ALS phenotypes.** Comparison of Nf between various clinical ALS phenotypes **(A–B)**. Comparison of BMD between various clinical ALS phenotypes **(C–F)**. Horizontal line shows median, whiskers illustrate interquartile range (0.25–0.75), and each icon represents an individual patient. Calculated by one-way ANCOVA considering ALS-PR as a covariate. Significance levels after Bonferroni-corrected *post hoc* analysis: *P < 0.05; ***P < 0.001. ALS, amyotrophic lateral sclerosis; ALS-PR, ALS progression rate; CK, creatine kinase; Crn, creatinine; Mb, myoglobin; Nf, neurofilaments; NfL, neurofilament light chain; pNfH, phosphorylated neurofilament heavy chain; PLS, primary lateral sclerosis; PMA, progressive muscular atrophy; TnT, troponin T.

After adjustment for ALS-PR as a confounding factor, Nf concentrations showed significant differences across the clinical phenotypes (NfL: *F*(2, 60) = 4.200, *P* = 0.020, partial *η*² = 0.123; pNfH: *F*(2, 73) = 10.575, *P* < 0.001, partial *η*² = 0.225). *Post hoc* analysis revealed that both Nf concentrations were significantly higher in patients with cALS compared to PMA [NfL: *P* = 0.018, Δmean = 0.664, 95% CI (0.09–1.24) and pNfH: *P* < 0.001, Δmean = 1.062, 95% CI (0.49–1.63)] (Fig. 3A and B). Of BMD, Mb and TnT concentrations also differed significantly regarding the clinical phenotypes (Mb: *F*(2, 45) = 3.316, *P* = 0.045, partial *η*² = 0.128; and TnT: *F*(2, 52) = 3.520, *P* = 0.037, partial *η*² = 0.119). *Post hoc* analysis revealed higher Mb concentrations in PMA than in PLS [*P* = 0.043, Δmean = 1.428, 95% CI (0.03–2.82)] (Fig. 3E). TnT levels were higher in PMA compared to PLS [*P* = 0.038, Δmean = 1.623, 95% CI (0.07–3.18)] and in cALS compared to PLS [*P* = 0.041, Δmean = 1.499, 95% CI (0.05–2.95)] (Fig. 3F). No significant differences were found for CK (*P* = 0.213) and Crn (*P* = 0.272) (Fig. 3C and D).

### Biomarkers in relation to LMN involvement

**Figure 4:**
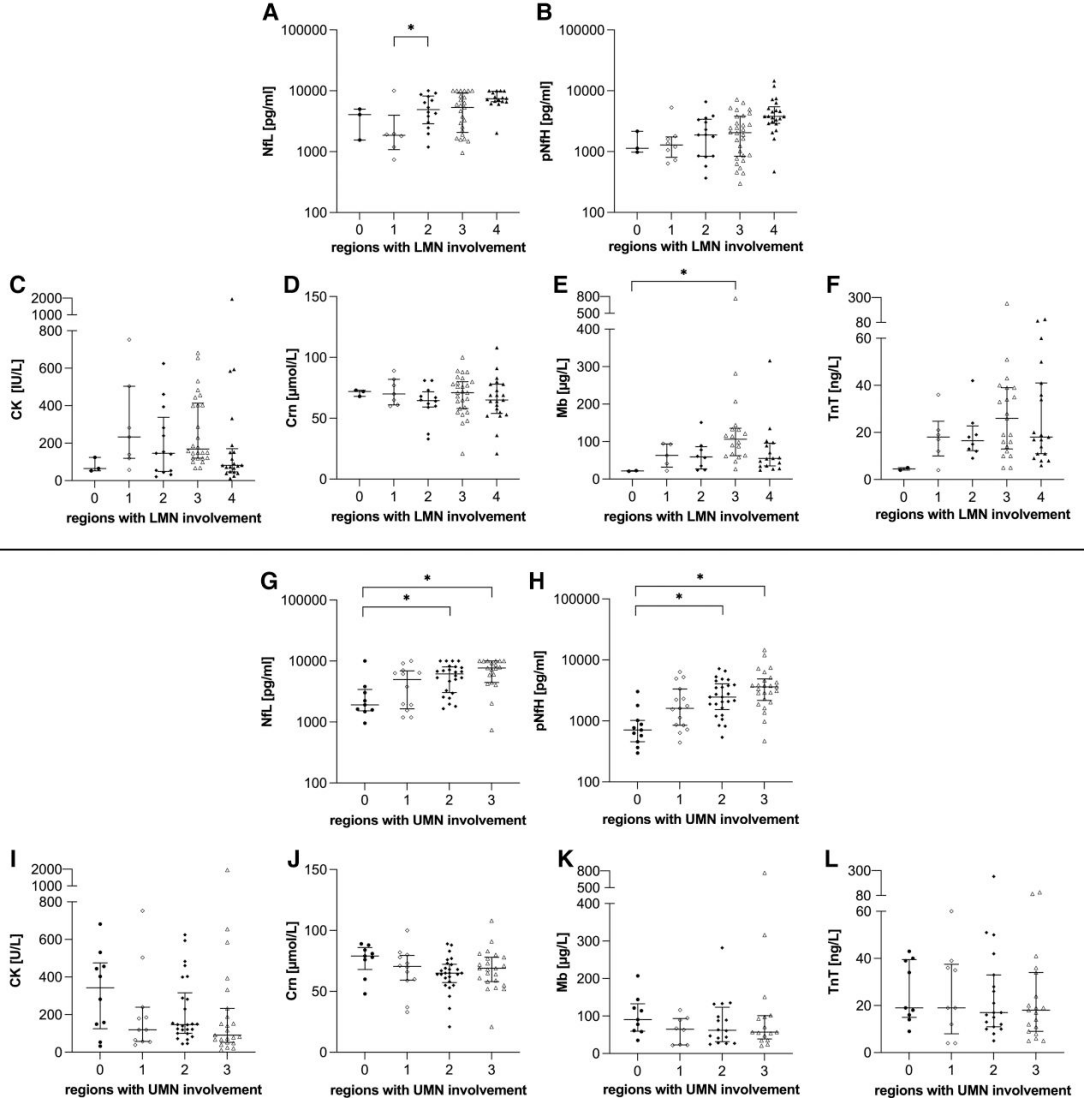
**Nf and BMD in relation to motor neuron involvement.** Comparison of Nf between subgroups of patients with ALS regarding LMN involvement **(A–B)**. Comparison of BMD between subgroups of patients with ALS regarding LMN involvement **(C–F)**. Comparison of Nf between subgroups of patients with ALS regarding UMN involvement **(G–H)**. Comparison of BMD between subgroups of patients with ALS regarding LMN involvement **(I–L)**. Horizontal line shows median, whiskers illustrate interquartile range (0.25–0.75), and each icon represents an individual patient. Calculated by one-way ANCOVA considering ALS-PR and UMN or LMN involvement as covariates. Significance levels after Bonferroni-corrected *post hoc* analysis: *P < 0.05. ALS, amyotrophic lateral sclerosis; ALS-PR, ALS progression rate; CK, creatine kinase; Crn, creatinine; LMN, lower motor neuron; Mb, myoglobin; NfL, neurofilament light chain; pNfH, phosphorylated neurofilament heavy chain; TnT, troponin T; UMN, upper motor neuron.

Considering the extent of LMN involvement, NfL showed slightly significant differences after adjustment for ALS-PR and UMN involvement (NfL: *F*(4, 57) = 2.835, *P* = 0.033, partial *η*² = 0.166). Patients with LMN involvement in 2 regions had significantly higher NfL concentrations than patients with LMN involvement in 1 region [*P* = 0.037, Δmean = 0.848, 95% CI 0.03–1.67)]. pNfH did not exhibit significant differences concerning the extent of LMN involvement (*P* = 0.618) (Fig. 4A and B).

For BMD, significant differences were only detected for Mb (*F*(4, 42) = 4.171, *P* = 0.006, partial *η*² = 0.284), revealing higher Mb levels in patients with LMN involvement in 3 regions compared to those without any LMN involvement [*P* = 0.014, Δmean = 1.749, 95% CI (0.24–3.26)] (Fig. 4E).

### Biomarkers in relation to UMN involvement

Investigating the extent of UMN involvement after adjustment for ALS-PR and LMN involvement, we observed significant differences for NfL (*F*(3, 58) = 4.152, *P* = 0.010, partial *η*² = 0.177) and pNfH [*F*(3, 71) = 8.777, *P* < 0.001, partial *η*² = 0.271]. *Post hoc* analyses revealed higher levels of both NfL and pNfH in patients with UMN involvement in 2 [NfL: *P* = 0.015, Δmean = 0.733, 95% CI (0.10–1.37); pNfH: *P* < 0.001, Δmean = 1.056, 95% CI (0.41–1.7)] and 3 regions [NfL: *P* = 0.024, Δmean = 0.736, 95% CI (0.06–1.41); pNfH: *P* < 0.001, Δmean = 1.172, 95% CI (0.5–1.85)] compared to those without any UMN involvement (Fig. 4G and H). No significant differences were detected for BMD regarding UMN involvement (Fig. 4I to L).

### Cluster analysis of biomarkers

**Figure 5:**
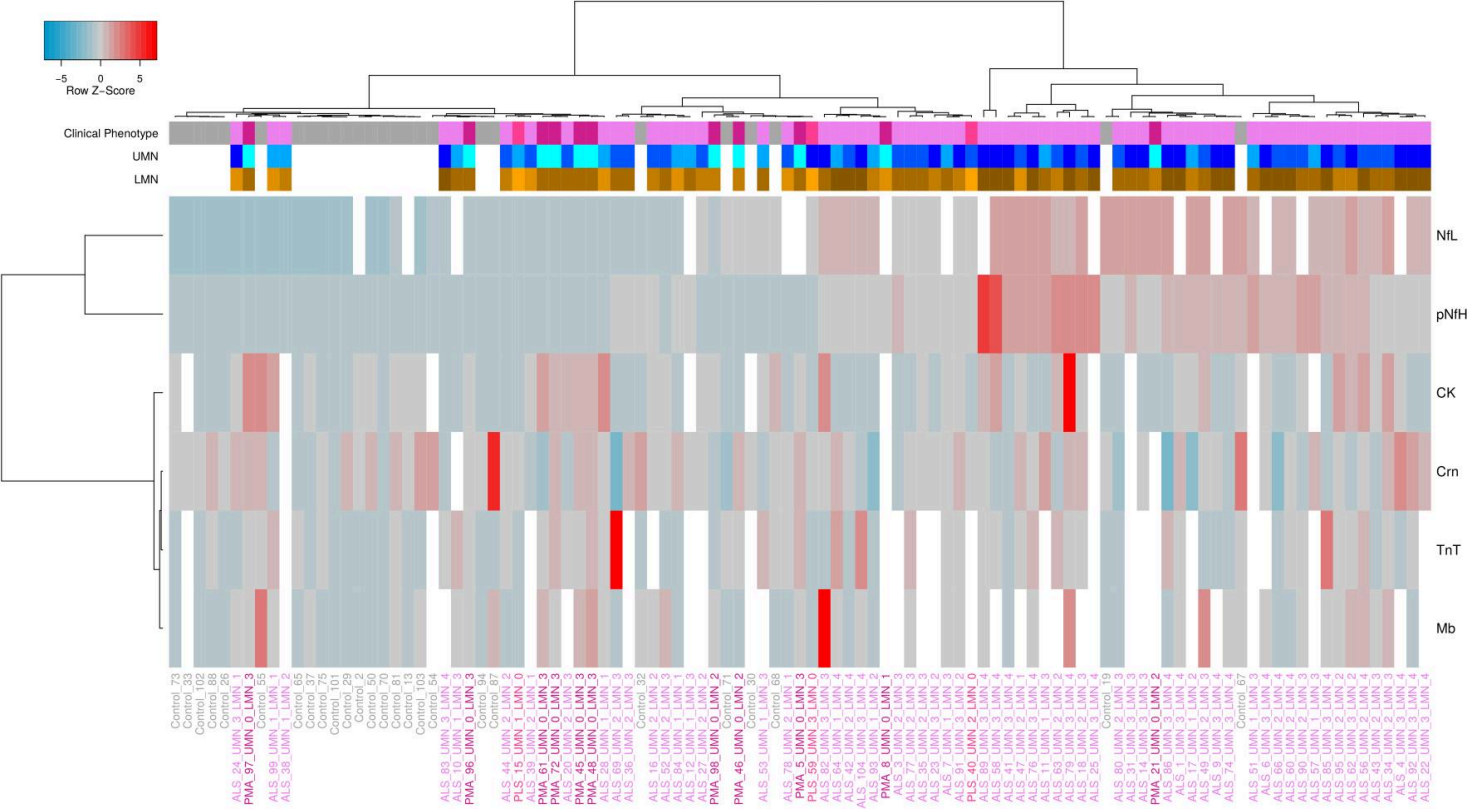
**Heatmap of biomarker measurements of 77 patients with ALS and clinical variants and 26 disease controls.** A colour gradient from blue over grey to red is used to visualize the Z-score scaled measurement of each biomarker across the patient and control samples. Missing biomarker values are displayed in white. The columns of the heatmap represent the individual patient and control samples with their corresponding clinical phenotype ALS (purple), PMA (violet), PLS (magenta), and control (grey) shown above the heatmap by the clinical phenotype bar. Corresponding states of the upper and lower motor neurons are shown by the UMN (blue shades, white: missing value) and LMN bars (orange shades, white: missing value). Darker shades correspond to more involved UMN and LMN regions, respectively. The rows of the heatmap represent the individual measurements of the biomarkers. The columns and rows of the heatmap were clustered hierarchically using Ward’s algorithm in combination with Euclidean distance to group similar samples and biomarkers together. ALS, amyotrophic lateral sclerosis; CK, creatine kinase; Crn, creatinine; LMN, lower motor neuron; Mb, myoglobin; NfL, neurofilament light chain; pNfH, phosphorylated neurofilament heavy chain; PLS, primary lateral sclerosis; PMA, progressive muscular atrophy; TnT, troponin T; UMN, upper motor neuron.

In a hierarchical cluster analysis, the vast majority of the study and disease control samples demonstrated distinct separation, with the disease controls primarily forming a separate cluster. The study cohort samples were distributed over three larger clusters with frequent co-clustering of samples with cALS and most of the samples with PMA. Co-clustering for PLS was not evident. The most discriminative markers between ALS and controls were NfL and pNfH. Patients with ALS or PMA, exhibiting comparable NfL and pNfH levels to those of disease controls, could be distinguished from disease controls based on elevated levels of BMD (Fig. 5).

### Biomarkers in relation to survival time

Of the ALS cohort, 34 patients (44%) died or underwent tracheostomy within the observation period of 70 months. The median time from disease onset to event (death or tracheostomy) was 29 months. The patients were classified into two groups, according to low (≤ median) and high (> median) levels of Nf and BMD.

**Figure 6:**
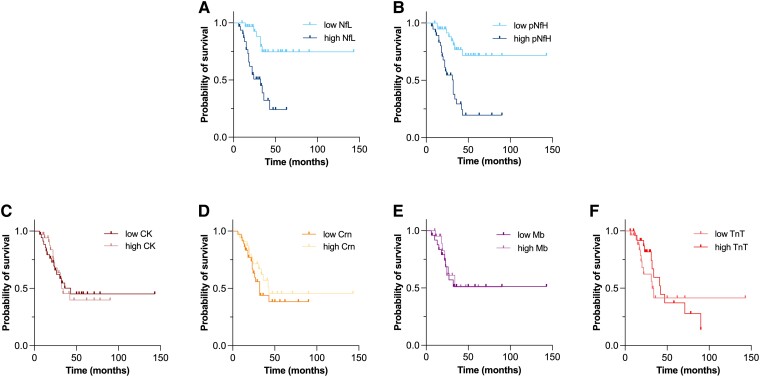
**Nf and BMD in relation to survival time.** Kaplan–Meier survival curves of Nf **(A–B)**. Kaplan–Meier survival curves of BMD **(C–F)**. Patients were classified into two groups (low with concentration ≤ median, high with concentration > median). Event was defined as death or tracheostomy. Kaplan–Meier method with log-rank test was performed for differences between both groups. BMD, biomarkers of muscular damage; CK, creatine kinase; Crn, creatinine; Mb, myoglobin; Nf, neurofilaments; NfL, neurofilament light chain; pNfH, phosphorylated neurofilament heavy chain; TnT, troponin T.

Patients with low NfL levels survived longer with a mean of 109 months [95% CI (87.17–130.17)] than patients with high NfL levels [mean: 30, 95% CI (22.87–38.09)]. Also, patients with low pNfH levels showed a longer mean survival time of 104 months [95% CI (83.4–123.83)] compared to patients with high pNfH levels [mean: 36, 95% CI (26.2–46.69)]. For both NfL and pNfH, survival distributions between the groups differed significantly [NfL: *χ²*(1) = 14.613, *P* < 0.001; pNfH: *χ²*(1) = 15.033, *P* < 0.001] (Fig. 6A and B). A subsequent Cox regression analysis of survival was performed for NfL and pNfH using age, site of onset, and ALS-PR as predicting survival covariates, respectively. We observed that high Nf levels were independently associated with poor survival (NfL: *P* = 0.012, OR = 3.285; pNfH: *P* = 0.019, OR = 2.332) (Supplementary Fig. 2). Concerning BMD, no associations with survival time were observed (Fig. 6C to F).

## Discussion

Nf and BMD have consistently demonstrated promise as biomarkers for ALS. However, comparative data emphasizing their strengths and weaknesses as well as their interpretation in daily care are deficient. In this cross-sectional monocentric study, we investigated and comprehensively compared Nf as biomarkers of neurodegeneration with CK, Crn, Mb, and TnT as biomarkers of muscular damage in relation to demographic and clinical features in patients with ALS and its clinical variants.

In line with published studies,^9,40,41^ both NfL and pNfH levels were elevated in patients with ALS compared to controls with ALS mimics and other neurodegenerative diseases. The obtained cut-off values of our study cohort were similar to published reference values.^9,42^ Diagnostic accuracy of pNfH was slightly higher than that of NfL, whereas both Nf strongly correlated with each other, as reported previously.^42,43^ Since Nf are especially abundant in large myelinated axons, levels are often highest in patients with ALS, as axonal damage contributes to a mass of release into CSF and blood.^5^

Concerning BMD, concentrations of Mb and TnT were also elevated in patients with ALS compared to disease controls. However, their substantive significance and diagnostic accuracy were inferior to that of Nf. Combined analyses obtained a higher sensitivity and specificity. As of our knowledge, there is limited data on Mb in the context of ALS. Having a crucial role in the oxygen-dependent metabolism of striated muscle fibres, we suggest that higher concentrations in ALS are presumably caused by secondary myopathy and subsequent release as previously hypothesized for other neuromuscular diseases.^44^ Our findings of elevated TnT levels in ALS confirm the results of a previous large cohort study.^30^ Elevated TnT concentrations are discussed as an upregulated re-expression in diseased muscle cells.^28,45^ Conclusively, TnT and Mb should rather be regarded as valuable supplementary biomarkers than confounders in the diagnosis of ALS. CK and Crn may also have discriminative potential for ALS, particularly in patients with non-elevated Nf values, as demonstrated by a cluster analysis. Although combined analyses of BMD obtained a higher sensitivity and specificity than BMD alone, their diagnostic accuracy was still inferior to that of Nf.

Reduced motor function, as assessed by the ALSFRS-R, was associated with elevated Nf concentrations, irrespective of age, sex, and disease duration. This implies that neuronal damage is related to disease severity in ALS. Rather moderate associating effects may be caused by a diagnostic ceiling effect for NfL in the assay used in this study, and by disease duration as a substantial confounder. Contrarily, elevated CK and Crn concentrations were associated with better motor function. As reported in ALS^20,46^ and adult patients with SMA,^21^ this finding most likely indicates the amount of preserved muscle mass.

Interestingly, as for Nf, we observed higher TnT concentrations associated with lower motor function. This is in line with recent findings in ALS and other neuromuscular diseases, propagating re-expression processes within striated muscle fibres subsequently leading to elevated TnT concentrations.^28,30,47^ While our results did not reveal significant associations between TnT and overall motor function, we did detect noticeable associations with impairment in subdomains specifically related to fine and gross motor functions. Mb did not show any relevant associations with motor functions. Considering disease progression, higher Nf concentrations indicated a faster disease progression, whereas BMD were not associated with progression rates in our cohort.

Investigating distinct clinical phenotypes of ALS is of great importance, as they vary in terms of survival expectancy, functional impairment, and medical demands.^3,4^ Thus, it will be important to explore the different biomarker profiles of these variants. We observed increased Nf, particularly pNfH, concentrations in classical ALS compared to PMA, in accordance with recent findings for serum NfL.^48^ As of our best knowledge, this is the first study systematically comparing BMD with regard to clinical ALS phenotypes and revealing higher Mb and TnT concentrations in PMA compared to PLS, independent of disease progression, presumably caused by the predominant muscle atrophy. These observations could also implicit a prognostic value in early disease stages: rather low Mb and TnT concentrations may be indicators for PLS, which is characterized by a slower disease progression with isolated UMN dysfunction for at least two years.^49^ Furthermore, a hierarchical cluster analysis unveiled the diagnostic potential of BMD, particularly CK, in distinguishing PMA from other diseases in patients with non-elevated Nf concentrations, underlining their complementary role in diagnosis.

Comparing biomarkers regarding the extent of motor neuron involvement, higher Nf concentrations were predominantly associated with the extent of UMN than with LMN involvement. As for NfL, this is consistent with previous findings, postulating a strong correlation between Nf concentrations and corticospinal tract degeneration.^50,51^ Interestingly, PLS with clinically pure UMN involvement showed lower Nf concentrations. This aligns with prior research, stating that pNfH concentrations are higher in classical ALS than in PLS.^52^ BMD concentrations showed no significant differences with regard to the extent of UMN involvement. Considering LMN involvement, we noted significant differences only for Mb, with non-significant subtle trends observed for TnT, thereby highlighting the pathomechanistic significance of BMD as markers for muscle atrophy caused by degeneration of LMN. However, these findings need to be validated in larger cohort studies.

In survival analysis, higher Nf concentrations were associated with shorter survival or time to tracheotomy. No relevant differences were observed between NfL and pNfH. Considering other prognostic predictors, Nf were confirmed to be independent prognostic factors. The prognostic value of BMD appears to be questionable. Higher CK and Crn concentrations are likely to be associated with longer survival in ALS.^22,24,46^ To our knowledge, there is inconsistent data regarding the prognostic significance of TnT^30,53^ and lacking survival research for Mb. Our observations did not confirm any notable correlation between BMD and survival prognosis.

The presented study includes some limitations, which warrant consideration in the interpretation of the findings. First, the retrospective and monocentric design of the study claims the potential for selection bias. Additionally, the absence of longitudinal assessments limits the ability to comprehensively evaluate biomarker dynamics related to disease progression and survival. Furthermore, the numerous missing data of BMD, especially of TnT and Mb, and the lack of serum NfL data may limit the applicability of our conclusions. Similarly, the sample sizes of the clinical phenotypes PMA and, particularly, PLS are relatively small, potentially comprising the statistical power of these subgroup analyses. Despite these limitations, our study contributes valuable insights into the domain of fluid biomarkers in ALS and underlines the need for further investigations with larger cohorts and comprehensive longitudinal assessments to validate and extend these findings.

## Conclusion

In the presented study, we comprehensively investigated and compared the diagnostic and prognostic potential of biomarkers of neurodegeneration and muscular damage in a ‘real-world’ cohort of patients with ALS and its clinical variants. Our data confirmed that Nf are reliable biomarkers to differentiate ALS from ALS mimics and other neurodegenerative diseases, monitor disease severity and progression, and predict survival. Nf may also be useful in distinguishing classical ALS from other clinical variants and phenotypes and is predominantly associated with UMN involvement. BMD are inferior to Nf in distinguishing ALS from other neuromuscular and neurodegenerative diseases, but demonstrate complementary utility, particularly when Nf levels are within normal range and for differentiating clinical ALS variants. CK, Crn, and TnT may serve as additional biomarkers of fine and gross motor function. In the context of survival or time to tracheostomy, only Nf exhibited prognostically relevant biomarker potential. Understanding the role of various biomarkers is crucial for enhancing diagnostics and monitoring novel therapies for ALS, underscoring the need for further investigation.

## Supplementary Material

fcae288_Supplementary_Data

## Data Availability

The datasets used and/or analysed during the current study are available from the corresponding author on reasonable request.
