# Supplementary material for: Comparative analysis of neurofilaments and biomarkers of muscular damage in amyotrophic lateral sclerosis
Source: Brain Commun. 2024 Aug 26;6(5):fcae288. doi: 10.1093/braincomms/fcae288 (PMC11375854; doi:10.1093/braincomms/fcae288)
Supplement: fcae288_Supplementary_Data [file fcae288_supplementary_data.docx]

**Supplementary material – Comparative analysis of Nf and BMD in ALS**

**Supplementary Fig. 1** **Neurofilaments and biomarkers of muscular damage in relation to disease progression**

**Supplementary Fig. 2 Neurofilaments and Cox regression analysis of survival**

**Supplementary Table 1** **Correlation analyses between neurofilaments and biomarkers of muscular damage and ALSFRS-R subscores and total score**

**Supplementary Table 2 Neurofilaments and biomarkers of muscular damage in relation to other clinical assessments**

**Supplementary Table 3 Neurofilaments and biomarkers of muscular damage in different clinical ALS phenotypes**


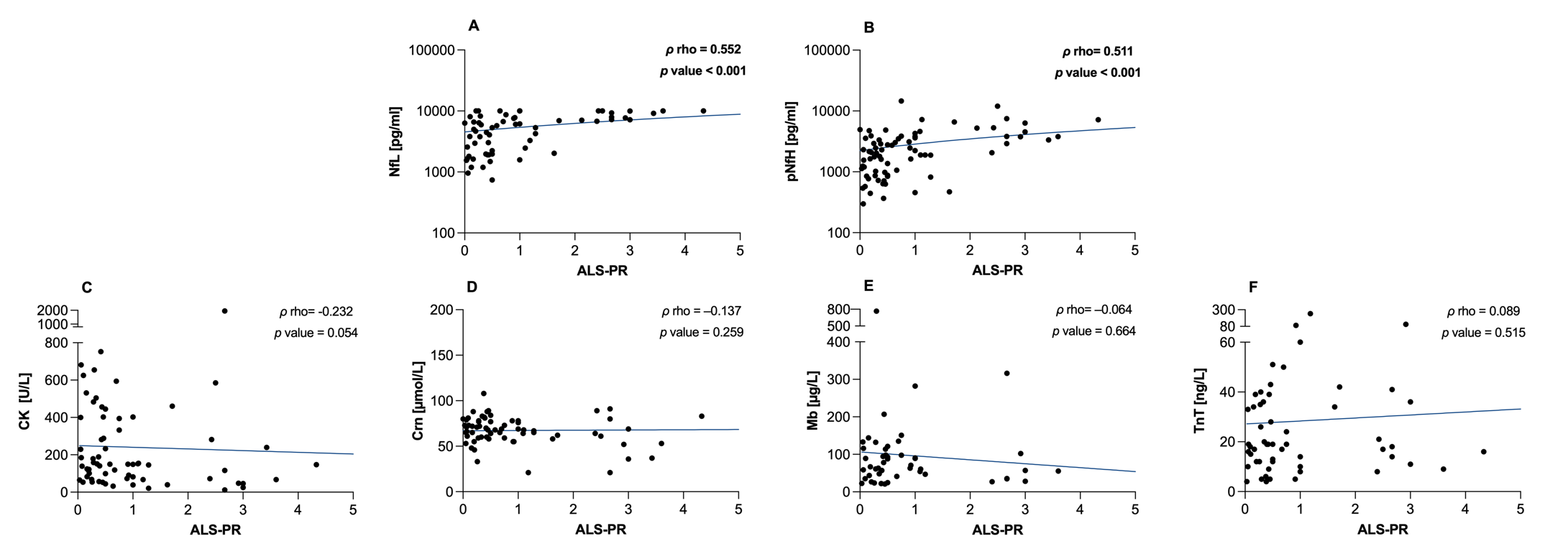


**Supplementary Fig. 1** **Neurofilaments and biomarkers of muscular damage in relation to disease progression.** Correlations between Nf and disease progression rate **(A–B)**. Correlations between BMD and disease progression rate **(C–F)**. Regression line in blue. Each icon represents an individual patient. Calculated by Spearman’s rank correlation. *P* values < 0.05 considered statistically significant and marked in bold. ALS, amyotrophic lateral sclerosis; ALS-PR, amyotrophic lateral sclerosis progression rate; CK, creatine kinase; Crn, creatinine; Mb, myoglobin; NfL, neurofilament light chain; pNfH, phosphorylated neurofilament heavy chain; TnT, troponin T

**
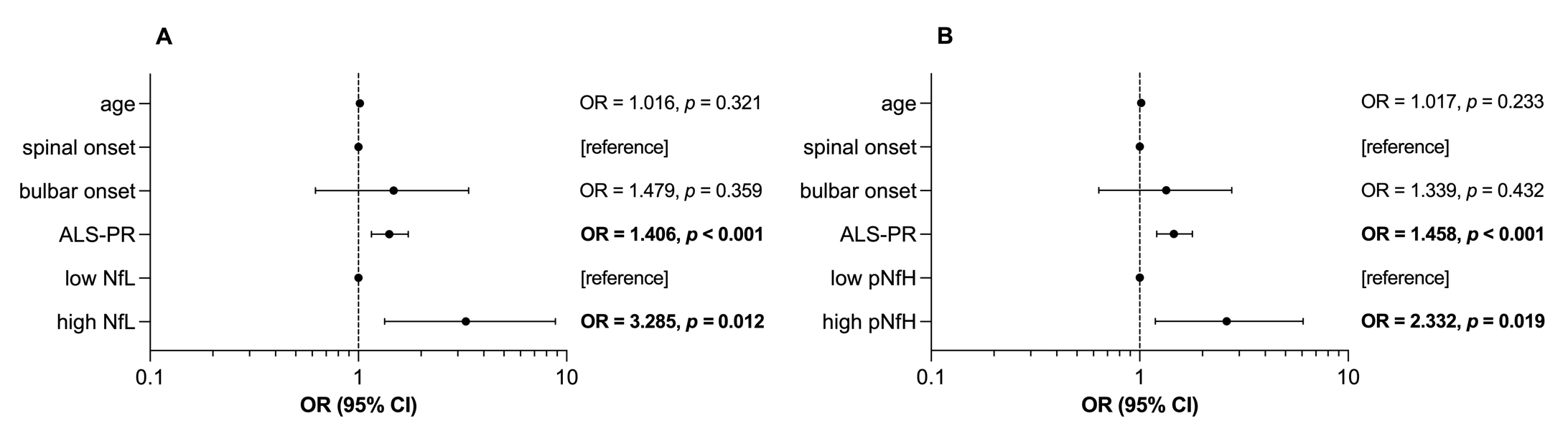
**

**Supplementary Fig. 2 Neurofilaments and Cox regression analysis of survival.** Cox regression analysis of survival for NfL **(A)** and pNfH **(B)**. ALS-PR, amyotrophic lateral sclerosis-progression rate; NfL, neurofilament light chain; pNfH, phosphorylated neurofilament heavy chain

**Supplementary Table 1 Correlation analyses between neurofilaments and biomarkers of muscular damage and ALSFRS-R subscores and total score**

|  | **NfL**  *n* = 64  d(f) = 59 | **adj *P*** | **pNfH**  *n* = 77  d(f) = 72 | **adj *P*** | **CK**  n = 70  d(f)=64 | **adj *P*** | **Crn**  n = 70  d(f)= 64 | **adj *P*** | **Mb**  n = 49  d(f) = 43 | **adj *P*** | **TnT**  n = 56  d(f) = 50 | **adj *P*** |
| --- | --- | --- | --- | --- | --- | --- | --- | --- | --- | --- | --- | --- |
| **ALSFRS-R bulbar functions [0 – 12]^a^** | *ρ* = –0.205;  *P* = 0.113 | 0.565 | *ρ* = –0.384;  ***P* < 0.001***** | **0.005**** | *ρ* = 0.228;  *P* = 0.066 | 0.33 | *ρ* = –0.050;  *P* = 0.693 | >0.99 | *ρ* = 0.239;  *P* = 0.113 | 0.565 | *ρ* = 0.160;  *P* = 0.258 | >0.99 |
| **ALSFRS-R fine motor functions [0 – 12]^a^** | *ρ* = –0.170;  *P* = 0.191 | 0.955 | *ρ* = –0.058;  *P* = 0.625 | > 0.99 | *ρ* = 0.214;  *P* = 0.084 | 0.42 | *ρ* = 0.329;  ***P* = 0.007**** | **0.035*** | *ρ* = 0.058;  *P* = 0.705 | >0.99 | *ρ* = –0.449;  ***P* < 0.001***** | **0.005**** |
| **ALSFRS-R gross motor functions [0 – 12]^a^** | *ρ* = –0.378;  ***P* = 0.003**** | **0.015*** | *ρ* = –0.151;  *P* = 0.198 | 0.99 | *ρ* = 0.304;  ***P* = 0.013** | 0.065 | *ρ* = 0.379;  ***P* < 0.001***** | **0.005**** | *ρ* = 0.031;  *P* = 0.841 | >0.99 | *ρ* = –0.380;  ***P* = 0.005**** | **0.025*** |
| **ALSFRS-R respiratory functions [0 – 12]^a^** | *ρ* = –0.293;  ***P* = 0.022*** | 0.11 | *ρ* = –0.212;  *P* = 0.070 | 0.35 | *ρ* = 0.420;  ***P* <0.001** | **0.005**** | *ρ* = 0.161;  *P* = 0.198 | 0.99 | *ρ* = 0.329;  ***P* = 0.027*** | 0.135 | *ρ* = –0.167;  *P* = 0.237 | >0.99 |
| **ALSFRS-R total score**  **[0 – 48]^a^** | *ρ* = –0.398;  ***P* = 0.002**** | **0.01*** | *ρ* = –0.335;  ***P* = 0.004**** | **0.02*** | *ρ* = 0.403;  ***P* <0.001** | **0.005**** | *ρ* = 0.378;  ***P* = 0.002**** | **0.01*** | *ρ* = 0.188;  *P* = 0.217 | >0.99 | *ρ* = –0.306;  ***P* = 0.028*** | 0.14 |

^a^Spearman’s partial rank correlation adjusted for sex, age, BMI, and disease duration. *ρ*, partial rank correlation coefficient, ** P* < 0.05, ** *P* < 0.01, *** *P* < 0.001. Adjusted *p* values for multiple testing after applying Bonferroni correction are given. ALS, amyotrophic lateral sclerosis; ALSFRS-R, Amyotrophic Lateral Sclerosis Functional Rating Scale – revised form; BMD, biomarkers of muscular damage; CK, creatine kinase; Crn, creatinine; Mb, myoglobin; NfL, neurofilament light chain; pNfH, phosphorylated neurofilament heavy chain; TnT, troponin T

**Supplementary Table 2 Neurofilaments and biomarkers of muscular damage in relation to other clinical assessments**

|  | **NfL [pg/ml]**  n = 64 | **pNfH [pg/ml]**  n = 77 | **CK [IU/L]**  n = 70 | **Crn [µmol/L]**  n = 70 | **Mb [µg/L]**  n = 49 | **TnT [ng/L]**  n = 56 |
| --- | --- | --- | --- | --- | --- | --- |
| **Disease duration [months]^a^** | ***ρ* = –0.471, *P* < 0.001***** | ***ρ* = –0.464, *P* < 0.001***** | *ρ* = –0.159, *P* = 0.189 | *ρ* = –0.141, *P* = 0.246 | *ρ* = –0.182, *P* = 0.210 | *ρ* = 0.151, *P* = 0.266 |
| **Site of onset**  **(spinal vs. bulbar)^b^** | *U* = 428.0, Z = –0.174,  *P* = 0.862 | *U* = 485.5, Z = –1.790,  *P* = 0.074 | *U* = 462.5, Z = –0.975,  *P* = 0.329 | *U* = 408.0, Z = –1.658,  *P* = 0.097 | *U* = 175.0, Z = –1.549,  *P* = 0.121 | ***U* = 174.5, Z = –2.641,**  ***P* = 0.008**** |
| **PEG**  **(yes or no)^b^** | *U* = 93.0, *Z* = –1.367,  *P* = 0.172 | *U* = 96.5, *Z* = –1.726,  *P* = 0.084 | *U* = 54.5, *Z* = –1.961,  *P* = 0.050 | *U* = 115.0, *Z* = –0.430,  *P* = 0.667 | *U* = 22.0, *Z* = –1.263,  *P* = 0.206 | ***U* = 7.0, *Z* = –2.077,**  ***P* = 0.038*** |
| **Assisted ventilation**  **(yes or no)^b^** | *U* = 172.0, *Z* = –1.462,  *P* = 0.144 | *U* = 225.5, *Z* = –1.659,  *P* = 0.097 | ***U* = 105.5, *Z* = –2.631,**  ***P* = 0.009**** | ***U* = 144.0, *Z* = –2.291,**  ***P* = 0.022*** | *U* = 129.0, *Z* = –0.947,  *P* = 0.344 | ***U* = 99.0, *Z* = –2.179,**  ***P* = 0.029*** |
| **Predicted FVC [%]^a^** | *ρ* = –0.258, *P* = 0.055 | *ρ* = –0.157, *P* = 0.200 | *ρ* = 0.002, *P* = 0.986 | *ρ* = 0.081, *P* = 0.535 | *ρ* = –0.199, *P* = 0.207 | *ρ* = 0.068, *P* = 0.646 |

^a^Spearman’s rank-order correlation. ^b^Mann–Whitney *U*-test. *ρ*, rank correlation coefficient, ** P* < 0.05, ** *P* < 0.01, *** *P* < 0.001. ALS, amyotrophic lateral sclerosis; CK, creatine kinase; Crn, creatinine; FVC, forced vital capacity; Mb, myoglobin; NfL, neurofilament light chain; pNfH, phosphorylated neurofilament heavy chain; PEG, percutaneous endoscopic gastrostomy; TnT, troponin

**Supplementary Table 3 Neurofilaments and biomarkers of muscular damage in different clinical ALS phenotypes**

|  | **cALS** | **PMA** | **PLS** |
| --- | --- | --- | --- |
| **NfL [pg/ml],**  median  (IQR)  range  above ULOQ, n (%) | n = 52  6642  (3413 – 9030)  738 – 10000  7 (14.5) | n = 9  1909  (1531 – 3417)  957 – 10000  1 (14.3) | n = 3  4073  (1552 – 5001)  1552 – 5001  0 (0.0) |
| **pNfH [pg/ml],**  median  (IQR)  range | n = 63  2883  (1623 ­– 4536)  444 – 14542 | n = 11  708  (456 – 1020)  297 ­– 3036 | n = 3  1131  (978 ­– 2160)  978 – 2160 |
| **CK [IU/L],**  median  (IQR)  range | n = 57  141  (73 – 285)  12 – 1952 | n = 10  342  (125 – 682)  32 - 682 | n = 3  65  (53 – 124)  53 – 124 |
| **Crn [µmol/L],**  median  (IQR)  range | n = 58  66  (58 – 77)  21 – 108 | n = 9  79  (68 – 89)  48 – 89 | n = 3  72.0  (68 – 73)  68 – 73 |
| **Mb [µg/L],**  median  (IQR)  range | n = 38  63  (40 – 99)  22 – 766 | n = 9  90  (60 – 133)  35 – 207 | n = 2  22  (21 – 23)  21 – 23 |
| **TnT [ng/L],**  median  (IQR)  range | n = 45  19  (12 – 36)  4 – 248 | n = 9  19  (15 – 40)  9 – 43 | n = 2  5  (4 – 5)  4 – 5 |

cALS, classical form of amyotrophic lateral sclerosis; BMD, biomarkers of muscular damage; CK, creatine kinase; Crn, creatinine; Mb, myoglobin; Nf, neurofilaments; NfL, neurofilament light chain; pNfH, phosphorylated neurofilament heavy chain; PLS, primary lateral sclerosis; PMA, progressive muscular atrophy; TnT, troponin
